# Selection of internal control genes for quantitative real-time RT-PCR studies during tomato development process

**DOI:** 10.1186/1471-2229-8-131

**Published:** 2008-12-22

**Authors:** Marino Expósito-Rodríguez, Andrés A Borges, Andrés Borges-Pérez, José A Pérez

**Affiliations:** 1 Instituto de Productos Naturales y Agrobiología – CSIC, Avda. Astrofísico Francisco Sánchez 3, P.O. Box 195, 38206 La Laguna, Tenerife, Canary Islands, Spain; 2 Departamento de Parasitología, Ecología y Genética – Facultad de Biología, Universidad de La Laguna, Avda. Astrofísico Francisco Sánchez s/n, 38271 La Laguna, Tenerife, Canary Islands, Spain

## Abstract

**Background:**

The elucidation of gene expression patterns leads to a better understanding of biological processes. Real-time quantitative RT-PCR has become the standard method for in-depth studies of gene expression. A biologically meaningful reporting of target mRNA quantities requires accurate and reliable normalization in order to identify real gene-specific variation. The purpose of normalization is to control several variables such as different amounts and quality of starting material, variable enzymatic efficiencies of retrotranscription from RNA to cDNA, or differences between tissues or cells in overall transcriptional activity. The validity of a housekeeping gene as endogenous control relies on the stability of its expression level across the sample panel being analysed. In the present report we describe the first systematic evaluation of potential internal controls during tomato development process to identify which are the most reliable for transcript quantification by real-time RT-PCR.

**Results:**

In this study, we assess the expression stability of 7 traditional and 4 novel housekeeping genes in a set of 27 samples representing different tissues and organs of tomato plants at different developmental stages. First, we designed, tested and optimized amplification primers for real-time RT-PCR. Then, expression data from each candidate gene were evaluated with three complementary approaches based on different statistical procedures. Our analysis suggests that SGN-U314153 (*CAC*), SGN-U321250 (*TIP41*), SGN-U346908 ("*Expressed*") and SGN-U316474 (*SAND*) genes provide superior transcript normalization in tomato development studies. We recommend different combinations of these exceptionally stable housekeeping genes for suited normalization of different developmental series, including the complete tomato development process.

**Conclusion:**

This work constitutes the first effort for the selection of optimal endogenous controls for quantitative real-time RT-PCR studies of gene expression during tomato development process. From our study a tool-kit of control genes emerges that outperform the traditional genes in terms of expression stability.

## Background

The study of gene expression patterns is one of the modern molecular biology cornerstones. Gene expression analyses have provided insight into complex biological processes, increasing our understanding of signalling and metabolic pathways that underlie environmental responses and development. Real-time reverse transcription PCR (real-time RT-PCR) is currently the standard method for accurate expression profiling of a moderate number of selected genes, its main advantages being a higher sensitivity and specificity, and a broader quantification range than previous molecular techniques [1-4]. Real-time RT-PCR analysis has become the most common method for verification of microarray expression results [5,6], reaching a notable level of throughput [7,8].

Regardless of the experimental technique employed, appropriate normalization is essential for obtaining an accurate and reliable quantification of gene expression levels, especially when measuring small expression differences or when working with tissues of different histological origin [9]. The purpose of normalization is to correct for variability associated with the various steps of the experimental procedure, such as differences in initial sample amount, RNA recovery, RNA integrity, efficiency of cDNA synthesis, and differences in the overall transcriptional activity of the tissues or cells analyzed. Among the numerous normalization approaches that have been proposed [10-15] the use of internal controls or reference genes has become the method of choice [3,4], because they potentially account for all the above-mentioned sources of variability. Since the internal control and target sequences are naturally present in the biological sample, both will undergo the same type of variation throughout the assay. The success of this normalization strategy is highly dependent on the choice of the appropriate control gene: its expression level should be relatively constant across the tissues or cells tested, and should not be significantly altered by the experimental pressures introduced [9]. If the expression of the reference gene is affected by an excessive variation the detection of small changes becomes unfeasible or, at worst, erroneous expression patterns could be inferred [16].

There is a general consensus on using housekeeping genes as internal controls in RT-PCR expression analyses. Since housekeeping genes are required for cellular survival, it is assumed that they are stably expressed and are often used without validating their suitability. However, numerous studies reported that the transcript levels of commonly used housekeeping genes can vary considerably under different experimental conditions [10,11,17-23]. Moreover, a reference gene with stable expression in one organism may not be suitable for normalization of gene expression in another [24,25]. In recent years, it has become clear that it is necessary to validate the expression stability of a candidate control gene in each experimental system prior to its use for normalization. In this regard, several free software-based applications such as geNorm [26], NormFinder [27] or qBase [28] permit the statistical identification of the best internal controls from a group of candidate normalization genes in a given set of biological samples. The combination of these statistical tools with microarray and expressed sequence tags (EST) data sets has been shown to be a valuable source of internal control genes for real-time RT-PCR experiments, providing a new generation of reference genes with very stable expression levels that outperform the classical housekeeping genes [23,27,29].

Tomato is an important model for genetic and molecular studies, and an international tomato genome project in currently in progress. However, a systematic study validating internal control genes for expression analyses of different developmental stages has not been accomplished in tomato as has occurred with Arabidopsis [23], rice [24] and soybean [25]. Searches of the literature reveal a single report in which several classical housekeeping genes are proposed as internal controls based on the relative abundance of tomato EST [30]. Nevertheless, no genes were identified that showed stable expression across a wide range of developmental conditions and any candidate control gene was further evaluated with a more accurate analytical technique. In the present report, we tested the performance of 7 classical and 4 novel housekeeping genes as internal controls for quantitative real-time RT-PCR experiments, in a set of 27 samples representing different tissues and organs of tomato plants at different developmental stages. In addition to 3 references genes suitable for transcript normalization in the whole developmental series, we recommended other combinations of internal controls that provide a more accurate normalization in studies focused on less heterogeneous sample panels.

## Results

### RNA quality

A set of 27 tissue samples from *Solanum lycopersicon *cv. ciliegia plants, comprising all tomato organs at different developmental stages, was processed with a commercial kit. Purified total RNAs had a mean value of 1.98 (SD = 0.09) for 260/280 nm ratios and showed, after denaturing electrophoresis, sharp and intense 18S and 25S ribosomal RNA bands with a practical absence of smears. The level of genomic DNA (gDNA) contamination in each RNA preparation was estimated by real-time PCR through the amplification of an alpha-tubulin gene sequence (tables 1 and 2). Only RNA samples from mature roots and immature green fruit gave a contamination signal, but with threshold cycle (Ct) values higher than 35. The cDNAs obtained from contaminated RNA samples were controlled during the corresponding RT-PCRs by means of reverse transcriptase-minus amplification reactions (RT-minus controls).

**Table 1:** Description of tomato candidate control genes

**Gene Symbol**	Tomato Accession Number*	**Arabidopsis**		
		
		**Homologous Locus**	**Locus Description/Function**	**Amino acid Identity with Tomato (%)**
GAPDH	U97257	AT1G13440	Glyceraldehyde-3-phosphate dehydrogenase/Glycolysis-Gluconeogenesis	91.9
EFα1	X53043	AT1G07940	Elongation factor 1-alpha/Translation elongation	67.5
TBP	SGN-U329249	AT1G55520	TATA binding protein/General RNA polymerase II transcription factor	94.8
RPL8	X64562	AT4G36130	Ribosomal protein L8/Structural constituent of ribosome	92.3
APT	BT012816	AT1G27450	Adenine phosphoribosyltransferase/Purine metabolism	84.4
DNAJ	AF124139	AT3G44110	DnaJ-like protein/Protein binding/folding	81.3
TUA	AC122540	AT5G19770	Alpha-tubulin/Structural constituent of cytoskeleton	92.0
TIP41	SGN-U321250	AT4G34270	TIP41-like family protein	67.5
SAND	SGN-U316474	AT2G28390	SAND family protein	61.4
CAC	SGN-U314153	AT5G46630	Clathrin adaptor complexes medium subunit/Endocytic pathway	95.0
Expressed	SGN-U346908	AT4G33380	Expressed sequence	66.4

* Accession numbers from GenBank database or Sol Genomics Network (SGN).

**Table 2:** Details of primers and amplicons for each of the 11 evaluated genes

**Gene Symbol**	**Oligo Sequence** **Forward/Reverse**	**Arabidopsis targeted Exons**	**Optimized** **Primer Concentration** **[μM]**	**Amplicon Length** **(pb)/Tm**		**Efficiency**	
					
				cDNA*	gDNA**	Mean***	**SD**
GAPDH	GGCTGCAATCAAGGAGGAA/AAATCAATCACACGGGAACTG	9th/10th 11th	0.2	207/78.1	N/A	0.913	0.027
EFα1	TACTGGTGGTTTTGAAGCTG/AACTTCCTTCACGATTTCATCATA	2nd 3rd	0.2	166/79.2	246/79.2	0.953	0.106
TBP	GCTAAGAACGCTGGACCTAATG/TGGGTGTGCCTTTCTGAATG	4th 6th	0.6	184/76.1	N/A	0.959	0.044
RPL8	CCGAAGGAGCTGTTGTTTGTA/ACCTGACCAATCATAGCACGA	1st 2nd	0.2	184/79.3	N/A	0.902	0.026
APT	CCATGAGGAAACCCAAGAAGT/CCTCCAGTCGCAATTAGATCAT	4th 5th	0.2	143/78.5	1150/75.2	0.887	0.024
DNAJ	GAGCACACATTGAGCCTTGAC/CTTTGGTACATCGGCATTCC	5th 6th	0.2	158/79.6	570/76.80	0.880	0.028
TUA	AGCTCATTAGCGGCAAAGAA/AGTACCCCCACCAACAGCA	2nd 3rd	0.2	163/77.0	254/78.60	0.973	0.106
TIP41	ATGGAGTTTTTGAGTCTTCTGC/GCTGCGTTTCTGGCTTAGG	6th/7th 8th	0.4	235/78.3	1157/80.2	0.941	0.053
SAND	TTGCTTGGAGGAACAGACG/GCAAACAGAACCCCTGAATC	6th 7th	0.4	164/78.2	N/A	0.944	0.053
CAC	CCTCCGTTGTGATGTAACTGG/ATTGGTGGAAAGTAACATCATCG	7th 8th	0.6	173/76.4	610/78.0	0.931	0.047
Expressed	GCTAAGAACGCTGGACCTAATG/TGGGTGTGCCTTTCTGAATG	6th 7th	0.2	183/76.0	285/76.80	0.874	0.037

* Predicted from tomato cDNA sequences; ** Estimated by agarose gel electrophoresis; *** Mean of 3 technical replicas; N/A = Non-amplification

### Performance of amplification primers

**Figure 1:**
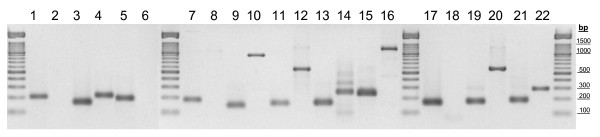
**Performance of the amplification primers**. Amplicons obtained by real-time PCR using cDNA (odd numbers) or gDNA (even numbers) as template, separated by agarose gel electrophoresis. Amplification primers were targeted to *GAPDH *(1–2), *EFα1 *(3–4), *TBP *(5–6), *RPL8 *(7–8), *APT *(9–10), *DNAJ *(11–12), *TUA *(13–14), *TIP41 *(15–16), *SAND *(17–18), *CAC *(19–20) and *Expressed (21–22) *tomato genes.

A total of 11 genes were selected as candidates for normalization of gene expression measures during tomato development studies (table 1). These include 7 classical (*GAPDH*, *EFα1*, *TBP*, *RPL8*, *APT*, *DNAJ *and *TUA*) and 4 novel (*TIP41*, *SAND*, *CAC *and SGN-U346908 – from now on referred to as "*Expressed*") housekeeping genes. In order to control for gDNA contaminations in the cDNA samples, PCR primers were designed on different exons (table 2) or spanning an exon-exon junction (forward primer for *GAPDH *and *TIP41 *genes), mainly guided by information about Arabidopsis genes. The performance of the amplification primers was tested by real-time PCR in two ways. First, aliquots from the 27 cDNA samples were pooled and used as template in amplification reactions with each primer-pair. A single band with the expected size (table 2) was obtained in each case without signs of primer-dimers formation (figure 1, odd lanes), as suggested by the previous melting curve analyses. Second, amplification primers were tested using gDNA as template (figure 1, even lanes). Seven primer-pairs yielded amplicons longer than those obtained with a cDNA template (table 2), whereas primers for *GAPDH*, *TBP*, *RPL8*, and *SAND *genes were unable to amplify genomic sequences. This result implies that intron position prediction in tomato genes was successful. As summarized in table 2, six amplicons obtained from gDNA have a melting temperature sufficiently different from those of corresponding cDNA amplicons to allow detection of gDNA interferences in a homogeneous assay. In the case of EFα1 primers, real-time RT-PCR should be followed by standard agarose gel electrophoresis. Absolute Tm values in table 2 should be considered with caution because they depend on the ionic strength of the actual reaction mix and the precision/accuracy of the real-time PCR platform.

Finally, in order to optimize PCR conditions, different primer concentrations were tested by real-time RT-PCR with the cDNA pool as template. Table 2 shows primer concentrations that provided the lowest Ct and thus the highest amplification efficiency.

### Ct data collection

Real-time RT-PCR was conducted on the 27 cDNA samples with the 11 primer-pairs. RT-minus controls were incorporated for mature roots and immature green fruit samples and only with the seven primer-pairs that yielded an amplification signal using gDNA as template. The 11 candidate control genes displayed a relatively wide range of expression level with mean Ct values between 21.1 (*GAPDH*) and 30.9 (*EFα1*). The RT-minus controls for mature roots and immature green fruit reached the fluorescence threshold only with APT, CAC and "Expressed" primers, but an extra treatment with DNase was not required because the Ct values of the mentioned RT-minus reactions were at least 10 cycles higher than those in the corresponding RT-PCRs, exceeding the minimum of 5 cycles recommended by Nolan *et al. *[3]. Amplification specificity was confirmed by melting analysis or, in the case of EFα1 primers, by agarose gel electrophoresis.

### Expression stability of housekeeping genes in the whole developmental series

In order to identify the most stably expressed genes during tomato development, the entire Ct dataset was analyzed using three different statistical approaches that have been incorporated in free specific VBA applets. The "pairwise comparison strategy" [26], implemented in the geNorm software, evaluates the variation of relative quantity (RQ) ratios for each gene-pair along the sample series. The "model-based approach for estimation of expression variation" [27], supported by the NormFinder software, estimates intra- and intergroup variation, and thus subdivision of the sample set in at least two coherent groups is required for the correct application of this approach. In this sense, we initially established the following sample-groups: roots (n = 5), leaves (n = 7; including cotyledons), inflorescences (n = 9) and fruits (n = 6). Since a minimum of 8 samples/group is recommended [27], expression data from different organs were also combined into "vegetative" (roots and leaves; n = 12) and "reproductive" (flowers and fruits; n = 15) sample-groups. The third statistical approach determines the expression stability for each control gene as the coefficient of variation (CV) of the relative expression levels after normalization. This evaluation strategy has been incorporated in the qBase program although limited to the analysis of 5 candidate genes [28].

The results of the three evaluation approaches are shown in table 3. It is noteworthy that definition of sample-groups had a notable effect on NormFinder output. Only two different NormFinder analyses were included in table 3 because we believe that sample grouping should not be arbitrary in an effort to adjust group sizes to increment statistical power, but rather that it reflects comparisons that researchers wish to make. It is remarkable that the NormFinder output with 4 sample-groups and CV ranking differ only in the relative position of the *CAC *and *TIP41 *genes. The results of the three statistical analyses exhibit several common features: *i*) *CAC *and *TIP41 *always rank as the most stably expressed housekeeping genes; *ii*), "*Expressed*", *TBP *and *SAND *also exhibit a remarkable stability of their expression levels and are always included among the 5 best performing reference genes; *iii*) *GAPDH, EFα1 *and *TUA *show unstable expression patterns and are always classified among the least reliable control genes.

**Table 3:** Ranking of the candidate control genes according to their expression stability in the whole developmental series.

**geNorm**	**NormFinder**		**Coefficient of Variation**	**Consensus**	
				
	2 groups	4 groups			
*CAC/TIP41*	*TIP41*	*TIP41*	*CAC*	*CAC/TIP41*	1
	*CAC*	*CAC*	*TIP41*		2
*TBP*	*SAND*	*Expressed*	*Expressed*	*Expressed*	3
*SAND*	*Expressed*	*TBP*	*TBP*	*TBP*	4
*Expressed*	*RPL8/TBP*	*SAND*	*SAND/RPL8*	*SAND*	5
*DNAJ*		*RPL8*		*RPL8*	6
*APT*	*APT*	*APT*	*APT*	*APT*	7
*RPL8*	*DNAJ*	*GAPDH/DNAJ*	*GAPDH*	*DNAJ*	8
*GAPDH*	*EFα1*		*DNAJ*	*GAPDH*	9
*EFα1*	*GAPDH*	*EFα1*	*EFα1*	*EFα1*	10
*TUA*	*TUA*	*TUA*	*TUA*	*TUA*	11

Expression data were evaluated with three different statistical approaches, and their outcomes were summarized in a consensus ranking. Expression stability decreases from top to bottom. Details on values of stability parameters are available as additional file 1.

Since the different statistical analyses applied to the expression data represent complementary strategies, we decided to combine results of the three evaluation approaches in a consensus rank, after averaging the two NormFinder outputs. For this purpose, genes were scored from 1 (most stable) to 11 (less stable) based on their relative position in each individual list. When two candidate genes are co-localized in a particular ranking (i.e., CV of the corresponding expression stability values ≤ 15%), both were scored with the average of the two consecutive positions. From the resulting consensus rank (table 3) it can be concluded that the best choice for normalization of expression measures in the entire developmental series are *CAC *and *TIP41 *genes, followed by "*Expressed*". Analysis of pairwise variation between two sequential normalization factors (NF) revealed that three genes are sufficient to calculate an accurate sample-specific NF as the geometric mean of their RQs. That is, the addition of a fourth control gene into the *CAC*/*TIP41*/*Expressed *combination does not significantly change NFs. The variation value for the pairs NF_3_/NF_4 _(V_3/4 _= 0.118) is lower than the default cut-off value of 0.15 [26]. The mean *M *and CV values for the *CAC*/*TIP41*/*Expressed *genes in the complete developmental series are M = 0.537 and CV = 0.338. These values are inside the ranges M ≤ 1 and CV ≤ 0.5, which have been proposed by Hellemans *et al*. [28] as acceptable for heterogeneous sample panels, such as the space-temporal one surveyed in the present study. Unfortunately, reference values for assessing the relevance of NormFinder scoring have not been specified by the software's authors [27]. In short, the *CAC*/*TIP41*/*Expressed *gene-triplet is recommended for accurate normalization of gene expression measures encompassing the complete development process in tomato.

**Figure 2:**
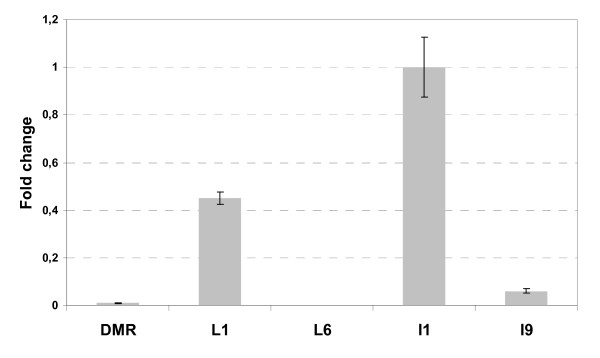
**Relative quantification of *ToFZY *mRNA in different tomato tissues**. Ct and amplification efficiency values were processed with the qBase software. Normalization factors were calculated as the geometric mean of the expression levels of three control genes (*CAC*, *TIP41 *and *Expressed*). The sample that showed the highest expression level was used as calibrator. cDNA samples came from the same set used in the evaluation of normalization: DMR, distal mature root; L1, younger leaf; L6, older leaf; I1, 1 mm bud; I9, open flower. Error bars show the standard deviation of three technical replicas.

To assess the validity of the procedure for the selection of control genes detailed above, the relative expression level of the *ToFZY *gene was estimated in five tomato tissue samples, using the control genes that we recommended for the normalization of gene expression measures in the whole developmental series. For this purpose, we used *ToFZY *specific primers described previously [31] and applied an efficiency-correction model for relative quantification [32]. Our results (figure 2) were highly concordant with the transcriptional pattern of *YUC1*/*YUC4 *genes (the Arabidopsis homologous to tomato *ToFZY *gene) reported by Cheng *et al*. [33] based on histochemical analysis of GUS reporter lines and *in situ *hybridization.

### Assessment of normalization in sample subsets

The same evaluation procedure applied to data from the whole developmental series was tested on different sample combinations which, in our understanding would represent plausible experimental contexts. Cotyledons were always included in the leaf sample group because top-ranked genes were not affected by this combination. The unique exceptions to the analysis routine were the inference of consensus gene-rankings for individual organs (root, leaf, inflorescence and fruit), which were constructed without participation of NormFinder software because an estimate of the intergroup variation is not possible. Results shown in table 4 can be used as a guide for selection of suitable control genes that fulfil specific research needs with regard to the particular developmental series analysed. The complete consensus rankings are available as additional file 1.

**Table 4:** Combinations of control genes recommended for different sample subsets.

**Sample subsets**	**Recommended Control genes**	**Mean stability values**		
			
		**M**	**CV**	V_n/n+1_*
R	*TIP41/SAND *(*CAC*)	0.299 (0.422)	0.210 (0.277)	0.149
L	*Expressed/TIP41 *(*CAC*)	0.399 (0.430)	0.246 (0.262)	0.135
I	*SAND*/*CAC *(*DNAJ*)	0.374 (0.416)	0.220 (0.236)	0.133
F	*CAC*/*SAND *(*Expressed*)	0.362 (0.306)	0.230 (0.256)	0.075
R+L	*TIP41*/*Expressed*/*CAC*	0.580	0.294	0.140
L+I	*CAC*/*Expressed*/*TIP41*	0.519	0.317	0.113
I+F	*CAC*/*SAND*/*RPL8*	0.507	0.307	0.123
L+I+F	*CAC*/*TIP41*/*Expressed*	0.481	0.343	0.109

For each sample subset, optimal control genes are displayed as arranged in the corresponding consensus ranking (see additional file 1). In brackets are shown optional control genes for individual organs and the resulting stability values (see comments in text). * Pairwise variation of NF_n_/NF_n+1 _ratios, *n *being the number of recommended control genes. R: roots; L: leaves and cotyledons; I: inflorescences; F: fruits.

The combinations of control genes recommended for the different sample subsets (table 4) are basically constructed with those that were ranked among the top five in the analysis of the whole developmental series (table 3), with the notable exception of *TBP *which is now downgraded in most consensus rankings (see additional file 1). It is clear that normalization of expression measures within organs have different requirements from comparisons between organs. On the one hand, two control genes are sufficient for accurate normalization in individual organs, as indicated by V_2/3 _values lower than 0.15 (table 4). The recommended gene-pairs have mean stability values that are acceptable (M ≤ 0.5 and CV ≤ 0.25) for relatively homogeneous sample panels [28]. In these cases, a third control gene is included (table 4, in brackets) for those wanting to use a minimum of 3 genes for calculating NFs, as suggested by Vandesompele *et al*. [26]. In addition, the *SAND *gene is revealed as appropriate for normalization within organs, but less advisable for between-organs experiments. On the other hand, when the sample subsets were comprised of 2 or 3 different organs the evaluation procedure indicated that 3 control genes are necessary for a reliable normalization (see V_3/4 _values in table 4). The expression levels of the 2 proposed triplets of control genes undergo oscillations comparable to those observed in the entire sample set, to judge by the values of the corresponding stability parameters (mean M and CV values in table 4). Moreover, control genes recommended for normalization in the complete developmental series (*CAC*/*TIP41*/*Expressed*) are also suitable for 3 different combinations of plant organs. The only exception is the subset integrated by inflorescence and fruit samples which can be suitably normalized with the following gene-triplet: *CAC*/*SAND*/*RPL8*. In this case, if *RPL8 *is substituted by *TIP41*, the next most stable gene in the inflorescence/fruit consensus ranking (additional file 1), the mean values for stability parameters would remain acceptable (M = 0.433 and CV = 0.341) and, at the same time, would allow the tool-kit of control genes for normalization during tomato development to be reduced to 4 components: *CAC*, *TIP41*, *Expressed *and *SAND*.

## Discussion

The detection of differentially expressed genes has contributed to understanding how developmental processes are conducted in a biological system such as tomato plant. In the field of gene-expression analysis, real-time reverse transcription PCR (RT-PCR) has become the method of choice for accurate expression profiling of selected genes [1-4]. Correct sample-normalization is an absolute prerequisite for reliable and accurate measurement of gene expression, especially when studying the biological relevance of small differences or when handling samples from different organs or tissues [9]. The actual gold standard for controlling inter-sample variations, both in the amount and quality of cDNA inputs, is the use of suitable genes as endogenous controls [3]. However, since there are no universal control genes, a set of potential references must be previously validated in each particular experimental background. Recently, an exhaustive analysis anchored on microarray data about expression profiles in Arabidopsis [23] allowed the identification of hundreds of potential reference genes, which show exceptional expression stability throughout development and under a wide range of environmental conditions. Despite its relevance as a model organism, certain biological processes are not tractable in Arabidopsis, such as the ripening of fleshy fruits which has received considerable attention in tomato. In addition, the conclusions derived from studies in Arabidopsis cannot be directly extrapolated to any vascular plant species. For example, *UBQ10 *gene shows highly stable expression in Arabidopsis [23], whereas it seems unsuitable for normalization in different tissues at different developmental stages in rice and soybean [24,25]. This emphasizes the importance of preliminary evaluation studies, aimed to identify the most stable housekeeping genes in different organisms. Taking the above-mentioned arguments into account, we accomplished a systematic study of the expression stability of 11 housekeeping genes in *Solanum lycopersicon*, along a series composed of 27 samples from different tissues/organs at different developmental stages.

In an effort to minimize bias introduced by the validation approach, three different, yet complementary, statistical strategies were used to select the best internal controls for normalization of gene expression studies in tomato. The pairwise comparison strategy, accessible through the geNorm software [26], is a very popular option for verifying the expression stability of candidate genes. It relies on the principle that variations in the expression ratios of two housekeeping genes reflect the fact that at least one of the two genes is not constantly expressed. Its main advantage is that expression ratios allow a fine control of variations in the amount of cDNA inputs, because these oscillations associated to technical variability affect both paired genes equally. It has been argued that the major weakness of the pairwise comparison approach is its sensitivity to co-regulation, that is, it apparently tends to select those genes with the highest degree of similarity in their expression profile [27]. However, it should be noted that the stability measure provided by geNorm (M) is the mean pairwise variation between a gene and all other tested candidates, and thus a pair of highly co-regulated genes could soon be eliminated during the selection process if they show high inter-sample variability. In addition, the advantage of two co-regulated genes is inversely proportional to the number of candidate genes being validated. An obvious prediction about behaviour of two co-regulated genes in the pairwise variation approach is that they will be scored with a similar M value. Indeed, there are numerous examples in the literature of genes belonging to the same functional class (typically different subunits of the same multiprotein complex) that are not top-ranked by the geNorm software, but which occupy closed positions in the ranking. Whatever that means, and since it is very difficult to foresee common expression patterns, the threshold cycle data were analyzed with two other statistical strategies that are less sensitive towards co-regulation of the candidate genes. On the one hand, the "model-based approach" implemented in the NormFinder software examines variation within and between sample groups that must be defined by the user. On the other hand, overall expression variation of each candidate gene was measured as the coefficient of variation (CV) of the normalized relative quantities (NRQ). The NormFinder approach stands out because it makes a balance of two sources of variation, but it does not account for systematic errors during sample preparation. Nevertheless, the CV strategy overcomes this drawback through the handling of normalized quantities, and may be a good alternative when the sample set cannot be appropriately subdivided. Although other valid statistical strategies have been successfully applied to control gene selection [34], the above-mentioned approaches are usually preferred because they are supported by user-friendly software.

Since the 3 statistical approaches complement one another their outcomes were equally weighted and combined into a consensus ranking. As the main result of this analysis, based on real-time PCR data, we proposed a tool-kit of control genes suitable for normalization of gene expression measures in a wide variety of samples in tomato. This tool-kit is composed of 4 housekeeping genes (*CAC*, *TIP41*, *Expressed *and *SAND*), which are recommended in different combinations depending on the sample origin (tables 3 and 4). Our analysis suggests that studies involving different tomato organs require at least 3 control genes for reliable and accurate normalization, while two control genes are sufficient for experiments within particular organs. The method of calculating a sample-specific normalization factor as the geometric mean of multiple carefully selected housekeeping genes [26] is currently the golden standard [3,12]. This approach has been adopted by many researchers and has been empirically and statistically validated [26,35-37]. Although the minimal use of three control genes has been proposed for the correct normalization of RT-PCR data [26] the actual optimal number of control genes should arise from a balance between economic considerations and accuracy, keeping in mind that normalization with multiple genes is less error-prone than single gene normalization [26,35-37].

Among the housekeeping genes evaluated in the present study, *DNAJ*, *GAPDH *and *TUA *genes have been previously described as "candidate controls" in tomato plants [30]. These genes were selected after the expression analysis of 127 transcripts in 27 expressed sequence tag libraries, but none of them was described as a suitable control gene for all tissues. Our results, based on data obtained with a more accurate and precise technique, lead to the conclusion that *DNAJ *gene may be useful for normalization in inflorescence samples (table 4) and, to a lesser extent, in leaves, fruits or a leaf/inflorescence developmental series (additional file 1). This is in accord with the results of Cocker and Davis [30]. However, we suggest that *GAPDH *and, especially, *TUA *should be avoided as control genes because their expression stability is far from acceptable. For instance, the NRQs of *TUA *gene showed CVs higher than 180% in leaf and fruit samples. As another contribution of the present report, our results indicate that reliable normalization of the whole tomato developmental series is possible with the *CAC*, *TIP41 *and *Expressed *genes. Finally, the results reported herein are in good agreement with those described in Arabidopsis by Czechowski *et al*. guided by microarray expression data [23]. In fact, the 4 control genes that we recommended for normalization in tomato are among the 5 top-ranked genes in Arabidopsis, although with a different relative position in the respective rankings. These novel control genes, as in Arabidopsis, are superior to traditional ones in terms of expression stability.

## Conclusion

This work constitutes the first in-depth study aimed to validate the optimal control genes for the quantification of transcript levels during tomato development using real-time RT-PCR technology. We have tested the expression stabilities of 11 candidate genes in a set of 27 tissue samples from tomato plants. As a result of this evaluation, we recommend 4 non-classical housekeeping genes as superior references for normalization of gene expression measures in different tomato developmental stages, and provide primer sequences whose performance in real-time PCR experiments is demonstrated. Finally, we have provided useful background information about the procedure of control gene selection in quantitative RT-PCR studies of gene expression.

## Methods

### Growth and Maintenance of Plants

Tomato (*Solanum lycopersicon*) cv. ciliegia plants were maintained under growth chamber conditions at 25 ± 2°C with standard potting compost in 9 cm diameter pots. The relative humidity was kept around 60% with a 12 h photoperiod (120 mol PAR m^-2 ^s^-1^). Plants were moved to a glasshouse when the 5-leaves stage was reached.

### Tissue collection

Sampling of the developmental series was prolonged over a 5-month period and comprised a total of 27 samples. The primary root that emerges through the seed coat was harvested at 72, 78 and 96 h following water imbibition. The proximal and distal portions of the mature root were collected at the 7-leaves stage. Cotyledons were excised at 96 h after seed imbibition. Six leaf samples were harvested per individual at the 6-leaves stage and always came from the apical leaflet. A total of 9 inflorescence developmental stages were established on the basis of bud sizes (8 samples; from 1 to 8 mm) and flower opening, as proposed by Brukhin *et al*. [38]. Seeds and pericarp were gently removed from fruits at 3 different developmental stages: immature green, breaker and red stages. After collection, samples were immediately frozen in liquid N_2 _and stored at -80°C until RNA extraction.

### Total RNA and genomic DNA isolation

Total RNA was purified from tissue samples using the Spectrum™ Plant Total RNA Kit and on-column DNase I digestion, following the manufacturer's recommendations (Sigma-Aldrich). The amount of starting sample was 50 mg and this required the pooling of tissues from 5–30 individuals depending on the size of the material recovered. RNA was quantified using absorbance at 260 nm, whereas its purity was assessed based on absorbance ratios at 260/280 nm. The integrity of purified RNA was confirmed by denaturing agarose gel electrophoresis and ethidium bromide staining. Genomic DNA was isolated from young leaves (100 mg) using the GenElute™ Plant Genomic DNA Miniprep Kit (Sigma-Aldrich) according to the manufacturer's instructions and checked by standard agarose electrophoresis.

### Selection of tomato sequences

We selected 11 potential reference genes (table 1) that belong to different functional classes to reduce the chance that the genes might be co-regulated, with the possible exception of *RPL8 *and *EFα1 *since both participated in the translation process. This group of genes comprised several classical housekeeping genes which are commonly used as internal control for expression studies [7,24,35,39,40], such as *GAPDH *(glyceraldehyde-3-phosphate dehydrogenase), *EFα1 *(elongation factor α1), *TBP *(TATA binding protein), *RPL8 *(ribosomal protein L8), *APT *(adenine phosphoribosyl transferase), *DNAJ *(DnaJ-like protein) and *TUA *(alpha-tubulin). Based on expressed sequence tag data, the *GAPDH*, *DNAJ *and *TUA *genes have been proposed as internal controls for expression analyses involving different tomato organs [30].

The set of candidate reference genes also included less conventional housekeeping genes which showed highly stable expression levels in analyses of microarray data-sets from Arabidopsis [23], such as *TIP41 *(TIP41-like protein), *SAND *(SAND family protein), *AT5G46630 *(clathrin adaptor complexes subunit) or *AT4G33380 *(expressed sequence). Some of these genes have also showed to be stably expressed in a variety of grapevine tissues [35]. Potential homologs to the corresponding Arabidopsis housekeeping genes were identified in the tomato unigen collection of the SOL Genomics Network (Cornell University; ) via amino acid sequence comparisons with the BLASTX application.

### PCR primer design

The amplification primers for real-time PCR were designed using PRIMER3 software [41]. In order to control for genomic DNA contamination, amplification primers were targeted to different exons (table 2). Information about exon positions in tomato *GAPDH *and *EFα1 *genes was directly available from databases. For the remaining tomato housekeeping genes, exon/intron boundaries were predicted through alignments [42] involving amino acid or nucleotide sequences from Arabidopsis and tomato, and based on information about exon positions from Arabidopsis Genome Project. The performance of the designed primers (table 2) was tested by real-time PCR using either tomato cDNA or genomic DNA templates.

### Real-time PCR

Real-time amplification reactions were performed using SYBR Green detection chemistry and run in triplicate on 96-wells plates with the iCycler iQ thermocycler (Bio-Rad). Reactions were prepared in a total volume of 20 μl containing: 4 μl of template, 2 μl of each amplification primer (optimized concentration in table 2), 10 μl of 2× FastStart SYBR Green Master (Roche Applied Science) and 2 μl of fluorescein as normalization dye. Blank controls were run in triplicate for each master mix. The cycling conditions were set as follows: initial denaturation step of 95°C for 10 min to activate the FastStart Taq DNA polymerase, followed by 45 cycles of denaturation at 95°C for 15 s, annealing at 60°C for 30 s and extension at 72°C for 30 s. The amplification process was followed by a melting curve analysis, ranging from 60°C to 90°C, with temperature increasing steps of 0.2°C every 10 s. Baseline and threshold cycles (Ct) were automatically determined using the Bio-Rad iQ Software 3.0.

The cDNA samples for real-time RT-PCR experiments were synthesized from 1 μg of total RNA and random nonamer primers, using the First-Strand Synthesis System of Sigma-Aldrich. The cDNAs were diluted to a final volume of 200 μl. A mixture of the 27 diluted cDNA samples was used for selecting the optimal concentration of each PCR primer pair (table 2), in a 0.2–0.6 μM range and based on the generation of lowest Ct values. The PCR efficiency was determined for each primer pair in its optimal concentration (table 2) with the DART-PCR workbook [43], which uses fluorescence data captured during the exponential phase of each amplification reaction. The amplicons obtained with each primer pair from the cDNA mixture and from a random subset of individual cDNA samples were checked by electrophoresis on 2% agarose gels and ethidium bromide staining.

The possibility of genomic DNA contamination in the RT-PCR assays was controlled in two ways, and through the ability of amplification primers to generate different amplicons from genomic DNA than from cDNA. First, each primer pair was tested by real-time PCR using tomato genomic DNA as template (1 ng). The melting temperature and the size of the amplicons obtained in these reactions were annotated (table 2) and considered further in the analyses of the RT-PCR results. Second, for each of the 27 RNA samples, a quantity equivalent to the cDNA used in the amplification reactions (i.e. 20 ng of total RNA) was amplified by real-time PCR using primers targeted to alpha-tubulin sequences (table 2). These primers provided a great power for detecting genomic DNA contaminations. RNA samples giving alfa-tubulin amplification were further controlled by means of RT-minus amplification reactions.

### Statistical analysis of gene expression stability

The suitability of candidate control genes was evaluated by applying three different statistical approaches to expression data (i.e. Ct values). These strategies provide complementary measures of gene expression stability among cDNA samples. In the first approach, Ct values were converted into relative quantities (RQs) using the sample with the lowest Ct as calibrator and taking into account the amplification efficiencies calculated for each primer-pair (table 2), and then imported into geNorm v.3.5 software [[26]; ]. This program estimates an expression stability value (*M*) for each gene, defined as the average pairwise variation of a particular gene with all other control genes in a given panel of cDNA samples. Genes with the lowest *M *values have the most stable expression. Housekeeping genes are ranked by geNorm through the elimination of the worst-scoring candidate control gene (this is, the one with the highest M value) and recalculating of new M values for the remaining genes. After this procedure is completed, two candidate genes are always top ranked because expression ratios are required for gene-stability measurements. The geNorm program also allows the minimal number of control genes required for calculating an accurate normalization factor (NF) to be determined, as the geometric mean of their RQs, but restricted to the gene ranking previously defined by the same program. This statistical procedure was adapted to any list of ordered genes in a homemade Excel worksheet. For this aim, first a NF is calculated for each sample with the two top ranked genes. Then, the most stable remaining gene is stepwise included and the NF is recalculated in each step. Finally, a pairwise variation of two sequential NF (V_n/n+1_) was estimated as the standard deviation of the logarithmically transformed NF_n_/NF_n+1_ratios, reflecting the effect of including an additional gene. A pairwise variation of 0.15 is accepted as cut-off [26], below which the inclusion of an additional control gene is not required for reliable normalization.

In the second evaluation approach, Ct values were log-transformed and imported into the NormFinder software [[27]; ]. This strategy is based on a mathematical model of gene expression that enables estimation of the intra- and intergroup variation, which are then combined into a stability value. Candidate control genes with the minimal intra- and intergroup variation will have the lowest stability value and will be top ranked. For adequate application of the NormFinder program, the sample set should be subdivided into at least two coherent groups, each one ideally integrated for a minimum of 8 samples.

In the third evaluation approach, the coefficient of variation of normalized relative expression levels was calculated for candidate genes throughout each developmental series tested. This statistical approach, proposed by Hellemans *et al*. [28], has been implemented in the qBase software  but only 5 candidate genes can be simultaneously evaluated. In order to overcome this limitation, we incorporated in an Excel worksheet the formulas 11, 13 and 15 described in [28] for calculating normalized relative quantities (NRQs). First, mean Ct values were transformed into RQs using the specific amplification efficiency of each primer-pair and the sample with the lowest Ct as calibrator (formula 11). Then, a sample-specific NF was calculated as the geometric mean of the RQs estimate for all candidate genes (formula 13). Finally, NRQs were calculated as the ratio of the RQ estimated for a gene/sample pair and the corresponding sample NF (formula 15).

## Authors' contributions

MER performed the entire experimental procedure; MER and JAP analyzed data and wrote the manuscript; MER, AAB, ABP and JAP conceived, designed and supervised the study. All authors read and critically revised the manuscript.

## Supplementary Material

Additional file 1 **Expression stability values and gene rankings for whole developmental series and different Samples subsets.** The data provide expression stability values and gene rankings for whole developmental series and different sample subsets. Click here for file
