# Supplementary material for: Selection of internal control genes for quantitative real-time RT-PCR studies during tomato development process
Source: BMC Plant Biol. 2008 Dec 22;8:131. doi: 10.1186/1471-2229-8-131 (PMC2629474; doi:10.1186/1471-2229-8-131)
Supplement: Additional file 1 — Expression stability values and gene rankings for whole developmental series and different Samples subsets. The data provide expression stability values and gene rankings for whole developmental series and different sample subsets. [file 1471-2229-8-131-S1.doc]

**Additional file1 : Expression stability values and gene rankings for whole developmental series and different Samples subsets**

Code: R: Roots; L: Leave + cotyledons; I: Inflorescences; F: Fruits

Genes that co-localized in a particular ranking are highlighted in bold

| **Samples: whole developmental series** | | | | | |  | | |  | | | | | |  |
| --- | --- | --- | --- | --- | --- | --- | --- | --- | --- | --- | --- | --- | --- | --- | --- |
| **geNorm** | | | **CV** | | | **NormFinder** | | | | | | | **Consensus** | |
|  |  |  |  |  |  | **2 sample-groups** | | | | **4 sample-groups** | | |  |  |  |
| **Gene Ranking** | **Stability value** | **Relative position** | **Gene Ranking** | **Stability value** | **Relative position** | **Gene Ranking** | **Stability value** | **Relative position** | | **Gene Ranking** | **Stability value** | **Relative position** | **Gene Ranking** | **Average**  **relative position** |
| **CAC** | 0.468 | 1.5 | CAC | 0.298 | 1 | TIP41 | 0.0017 | 1 | | TIP41 | 0.0061 | 1 | **CAC** | 1.5 |
| **TIP41** | 0.468 | 1.5 | TIP41 | 0.315 | 2 | CAC | 0.0018 | 2 | | CAC | 0.0080 | 2 | **TIP41** | 1.5 |
| TBP | 0.520 | 3 | Expressed | 0.403 | 3 | SAND | 0.0026 | 3 | | Expressed | 0.0082 | **3** | Expressed | 3 |
| SAND | 0.540 | 4 | TBP | 0.423 | 4 | Expressed | 0.0027 | 4 | | TBP | 0.0087 | **4** | TBP | 4 |
| Expressed | 0.564 | 5 | **SAND** | 0.440 | 5.5 | **RPL8** | 0.0030 | 5.5 | | SAND | 0.0090 | 5 | SAND | 5 |
| DNAJ | 0.646 | 6 | **RPL8** | 0.449 | 5.5 | **TBP** | 0.0030 | 5.5 | | RPL8 | 0.0102 | 6 | RPL8 | 6 |
| APT | 0.693 | 7 | APT | 0.490 | 7 | APT | 0.0032 | 7 | | APT | 0.0126 | 7 | APT | 7 |
| RPL8 | 0.749 | 8 | GAPDH | 0.547 | 8 | DNAJ | 0.0039 | 8 | | **GAPDH** | 0.0139 | 8.5 | DNAJ | 8 |
| GAPDH | 0.825 | 9 | DNAJ | 0.571 | 9 | EFa1 | 0.0049 | 9 | | **DNAJ** | 0.0142 | 8.5 | GAPDH | 9 |
| EFa1 | 0.898 | 10 | EFa1 | 0.738 | 10 | GAPDH | 0.0054 | 10 | | EFa1 | 0.0165 | 10 | EFa1 | 10 |
| TUA | 1.335 | 11 | TUA | 1.566 | 11 | TUA | 0.0147 | 11 | | TUA | 0.0298 | 11 | TUA | 11 |

| **Samples: R** | | |  | | |  | |
| --- | --- | --- | --- | --- | --- | --- | --- |
| **geNorm** | | | **CV** | | | **Consensus** | |
| **Gene Ranking** | **Stability value** | **Relative position** | **Gene Ranking** | **Stability value** | **Relative position** | **Gene Ranking** | **Average**  **relative position** |
| **RPL8** | 0.315 | 1.5 | TIP41 | 0.143 | 1 | TIP41 | 1 |
| **Expressed** | 0.315 | 1.5 | CAC | 0.244 | 2 | SAND | 2 |
| SAND | 0.468 | 3 | SAND | 0.277 | 3 | **CAC** | 3.5 |
| TIP41 | 0.503 | 4 | TBP | 0.406 | 4 | **RPL8** | 3.5 |
| CAC | 0.569 | 5 | **RPL8** | 0.460 | 5.5 | Expressed | 5 |
| TBP | 0.628 | 6 | **APT** | 0.463 | 5.5 | TBP | 6 |
| APT | 0.734 | 7 | DNAJ | 0.496 | 7 | APT | 7 |
| GAPDH | 0.788 | 8 | Expressed | 0.510 | 8 | DNAJ | 8 |
| DNAJ | 0.836 | 9 | GAPDH | 0.613 | 9 | GAPDH | 9 |
| TUA | 0.951 | 10 | TUA | 0.712 | 10 | TUA | 10 |
| EFα1 | 1.057 | 11 | EFα1 | 0.833 | 11 | EFα1 | 11 |

| **Samples: L** | | |  | | |  | |  |
| --- | --- | --- | --- | --- | --- | --- | --- | --- |
| **geNorm** | | | **CV** | | | **Consensus** | |
| **Gene Ranking** | **Stability value** | **Relative position** | **Gene Ranking** | **Stability value** | **Relative position** | **Gene Ranking** | **Average**  **Relative position** |
| **CAC** | 0.378 | 1.5 | TIP41 | 0.210 | 1 | Expressed | 1 |
| **Expressed** | 0.378 | 1.5 | Expressed | 0.282 | 2 | TIP41 | 2 |
| TIP41 | 0.431 | 3 | **APT** | 0.289 | 3.5 | CAC | 3 |
| TBP | 0.507 | 4 | **CAC** | 0.295 | 3.5 | APT | 4 |
| SAND | 0.549 | 5 | DNAJ | 0.308 | 5 | **DNAJ** | 5.5 |
| DNAJ | 0.615 | 6 | RPL8 | 0.384 | 6 | **TBP** | 5.5 |
| APT | 0.682 | 7 | TBP | 0.517 | 7 | SAND | 7 |
| RPL8 | 0.752 | 8 | SAND | 0.597 | 8 | RPL8 | 8 |
| GAPDH | 0.841 | 9 | GAPDH | 0.636 | 9 | GAPDH | 9 |
| EFα1 | 0.926 | 10 | EFα1 | 0.814 | 10 | EFα1 | 10 |
| TUA | 1.311 | 11 | TUA | 1.430 | 11 | TUA | 11 |

| **Samples: I** | | |  | | |  | |
| --- | --- | --- | --- | --- | --- | --- | --- |
| **geNorm** | | | **CV** | | | **Consensus** | |
| **Gene Ranking** | **Stability value** | **Relative position** | **Gene Ranking** | **Stability value** | **Relative position** | **Gene Ranking** | **Average**  **relative position** |
| **CAC** | 0.374 | 1.5 | SAND | 0.205 | 1 | SAND | 1 |
| **SAND** | 0.374 | 1.5 | CAC | 0.236 | 2 | CAC | 2 |
| DNAJ | 0.416 | 3 | **DNAJ** | 0.268 | 3.5 | DNAJ | 3 |
| RPL8 | 0.468 | 4 | **RPL8** | 0.270 | 3.5 | RPL8 | 4 |
| APT | 0.503 | 5 | TIP41 | 0.309 | 5 | **APT** | 5.5 |
| TIP41 | 0.537 | 6 | APT | 0.340 | 6 | **TIP41** | 5.5 |
| TBP | 0.564 | 7 | EFα1 | 0.420 | 7 | **EFα1** | 7.5 |
| Expressed | 0.604 | 8 | Expressed | 0.459 | 8 | **Expressed** | 7.5 |
| EFα1 | 0.639 | 9 | GAPDH | 0.464 | 9 | TBP | 9 |
| GAPDH | 0.672 | 10 | TBP | 0.497 | 10 | GAPDH | 10 |
| TUA | 1.001 | 11 | TUA | 1.090 | 11 | TUA | 11 |

| **Samples: F** | | |  | | |  | |  |
| --- | --- | --- | --- | --- | --- | --- | --- | --- |
| **geNorm** | | | **CV** | | | **Consensus** | |
| **Gene Ranking** | **Stability value** | **Relative position** | **Gene Ranking** | **Stability value** | **Relative position** | **Gene Ranking** | **Average**  **relative position** |
| **Expressed** | 0.204 | 1.5 | CAC | 0.170 | 1 | CAC | 1 |
| **SAND** | 0.204 | 1.5 | TBP | 0.181 | 2 | SAND | 2 |
| CAC | 0.306 | 3 | SAND | 0.291 | 3 | Expressed | 3 |
| APT | 0.384 | 4 | Expressed | 0.306 | 4 | **APT** | 5 |
| DNAJ | 0.400 | 5 | DNAJ | 0.325 | 5 | **DNAJ** | 5 |
| RPL8 | 0.431 | 6 | APT | 0.338 | 6 | **TBP** | 5 |
| TIP41 | 0.467 | 7 | **RPL8** | 0.351 | 7.5 | RPL8 | 7 |
| TBP | 0.492 | 8 | **TIP41** | 0.357 | 7.5 | TIP41 | 8 |
| EFα1 | 0.566 | 9 | EFα1 | 0.408 | 9 | EFα1 | 9 |
| GAPDH | 0.689 | 10 | GAPDH | 0.480 | 10 | GAPDH | 10 |
| TUA | 1.045 | 11 | TUA | 2.080 | 11 | TUA | 11 |

| **Samples: R+L** | | | | | |  | | |  | |
| --- | --- | --- | --- | --- | --- | --- | --- | --- | --- | --- |
| **geNorm** | | | **CV** | | | **NormFinder** | | | **Consensus** | |
| **Gene Ranking** | **Stability value** | **Relative position** | **Gene Ranking** | **Stability value** | **Relative position** | **Gene Ranking** | **Stability value** | **Relative position** | **Gene Ranking** | **Average**  **relative position** |
| **TBP** | 0.513 | 1.5 | TIP41 | 0.198 | 1 | TIP41 | 0.0033 | 1 | TIP41 | 1 |
| **SAND** | 0.513 | 1.5 | CAC | 0.296 | 2 | Expressed | 0.0052 | 2 | Expressed | 2 |
| **TIP41** | 0.582 | 3.5 | Expressed | 0.388 | 3 | **TBP** | 0.0081 | 3.5 | **CAC** | 3.5 |
| **Expressed** | 0.592 | 3.5 | **APT** | 0.449 | 5 | **CAC** | 0.0082 | 3.5 | **TBP** | 3.5 |
| CAC | 0.607 | 5 | **SAND** | 0.457 | 5 | SAND | 0.0089 | 5 | SAND | 5 |
| DNAJ | 0.710 | 6 | **TBP** | 0.463 | 5 | APT | 0.0091 | 6 | APT | 6 |
| APT | 0.750 | 7 | DNAJ | 0.499 | 7 | **GAPDH** | 0.0106 | 7.5 | DNAJ | 7 |
| GAPDH | 0.826 | 8 | RPL8 | 0.538 | 8 | **DNAJ** | 0.0115 | 7.5 | GAPDH | 8 |
| RPL8 | 0.894 | 9 | GAPDH | 0.602 | 9 | RPL8 | 0.0151 | 9 | RPL8 | 9 |
| EFα1 | 1.022 | 10 | EFα1 | 0.937 | 10 | EFα1 | 0.0160 | 10 | EFα1 | 10 |
| TUA | 1.291 | 11 | TUA | 1.340 | 11 | TUA | 0.0189 | 11 | TUA | 11 |

| **Samples: L+I** | | | | | |  | | |  | |
| --- | --- | --- | --- | --- | --- | --- | --- | --- | --- | --- |
| **geNorm** | | | **CV** | | | **NormFinder** | | | **Consensus** | |
| **Gene Ranking** | **Stability value** | **Relative position** | **Gene Ranking** | **Stability value** | **Relative position** | **Gene Ranking** | **Stability value** | **Relative position** | **Gene Ranking** | **Average**  **relative position** |
| **CAC** | 0.479 | 1.5 | CAC | 0.270 | 1 | CAC | 0.0044 | 1 | CAC | 1 |
| **Expressed** | 0.479 | 1.5 | DNAJ | 0.283 | 2 | Expressed | 0.0046 | 2 | Expressed | 2 |
| TIP41 | 0.519 | 3 | TIP41 | 0.299 | 3 | DNAJ | 0.0056 | 3 | TIP41 | 3 |
| TBP | 0.538 | 4 | Expressed | 0.382 | 4 | TIP41 | 0.0063 | 4 | DNAJ | 4 |
| SAND | 0.564 | 5 | APT | 0.407 | 5 | SAND | 0.0082 | 5 | TBP | 5 |
| DNAJ | 0.607 | 6 | RPL8 | 0.427 | 6 | TBP | 0.0085 | 6 | SAND | 6 |
| RPL8 | 0.666 | 7 | TBP | 0.514 | 7 | RPL8 | 0.0096 | 7 | RPL8 | 7 |
| APT | 0.721 | 8 | **SAND** | 0.534 | 8.5 | **GaPDH** | 0.0104 | 8.5 | APT | 8 |
| GAPDH | 0.775 | 9 | **GAPDH** | 0.540 | 8.5 | **APT** | 0.0106 | 8.5 | GAPDH | 9 |
| EFα1 | 0.833 | 10 | EFα1 | 0.814 | 10 | EFα1 | 0.0126 | 10 | EFα1 | 10 |
| TUA | 1.207 | 11 | TUA | 1.249 | 11 | TUA | 0.0219 | 11 | TUA | 11 |

| **Samples: I+F** | | | | | |  | | |  | |
| --- | --- | --- | --- | --- | --- | --- | --- | --- | --- | --- |
| **geNorm** | | | **CV** | | | **NormFinder** | | | **Consensus** | |
| **Gene Ranking** | **Stability value** | **Relative position** | **Gene Ranking** | **Stability value** | **Relative position** | **Gene Ranking** | **Stability value** | **Relative position** | **Gene Ranking** | **Average**  **relative position** |
| **CAC** | 0.366 | 1.5 | RPL8 | 0.296 | 1 | RPL8 | 0.0039 | 1 | **CAC** | 1.5 |
| **SAND** | 0.366 | 1.5 | CAC | 0.307 | 2 | SAND | 0.0076 | 2 | **SAND** | 1.5 |
| TIP41 | 0.433 | 3 | SAND | 0.317 | 3 | CAC | 0.0082 | 3 | RPL8 | 3 |
| TBP | 0.478 | 4 | **TIP41** | 0.398 | 4.5 | TIP41 | 0.0092 | 4 | TIP41 | 4 |
| Expressed | 0.509 | 5 | **TBP** | 0.401 | 4.5 | TBP | 0.0095 | 5 | TBP | 5 |
| DNAJ | 0.547 | 6 | **Expressed** | 0.413 | 6.5 | Expressed | 0.0105 | 6 | Expressed | 6 |
| APT | 0.565 | 7 | **APT** | 0.418 | 6.5 | APT | 0.0110 | 7 | APT | 7 |
| RPL8 | 0.592 | 8 | GAPDH | 0.478 | 8 | EFα1 | 0.0135 | 8 | DNAJ | 8 |
| EFα1 | 0.637 | 9 | EFα1 | 0.522 | 9 | DNAJ | 0.0155 | 9 | EFα1 | 9 |
| GAPDH | 0.733 | 10 | DNAJ | 0.564 | 10 | GPDH | 0.0168 | 10 | GAPDH | 10 |
| TUA | 1.305 | 11 | TUA | 1.551 | 11 | TUA | 0.0498 | 11 | TUA | 11 |

| **Samples: L+I+F** | | | | | |  | | |  | |
| --- | --- | --- | --- | --- | --- | --- | --- | --- | --- | --- |
| **geNorm** | | | **CV** | | | **NormFinder** | | | **Consensus** | |
| **Gene Ranking** | **Stability value** | **Relative position** | **Gene Ranking** | **Stability value** | **Relative position** | **Gene Ranking** | **Stability value** | **Relative position** | **Gene Ranking** | **Relative position** |
| **Expressed** | 0.440 | 1.5 | CAC | 0.298 | 1 | **CAC** | 0.0071 | 1.5 | CAC | 1 |
| **CAC** | 0.440 | 1.5 | TIP41 | 0.340 | 2 | **TIP41** | 0.0072 | 1.5 | **Expressed** | 2.5 |
| TBP | 0.484 | 3 | Expressed | 0.389 | 3 | Expressed | 0.0080 | 3 | **TIP41** | 2.5 |
| TIP41 | 0.505 | 4 | RPL8 | 0.414 | 4 | SAND | 0.0086 | 4 | TBP | 4 |
| SAND | 0.529 | 5 | TBP | 0.435 | 5 | TBP | 0.0089 | 5 | SAND | 5 |
| DNAJ | 0.601 | 6 | SAND | 0.466 | 6 | RPL8 | 0.0097 | 6 | RPL8 | 6 |
| APT | 0.659 | 7 | APT | 0.502 | 7 | EFα1 | 0.0131 | 7 | APT | 7 |
| RPL8 | 0.711 | 8 | GAPDH | 0.546 | 8 | APT | 0.0135 | 8 | DNAJ | 8 |
| EFα1 | 0.771 | 9 | DNAJ | 0.600 | 9 | **DNAJ** | 0.0148 | 9.5 | EFα1 | 9 |
| GAPDH | 0.847 | 10 | EFα1 | 0.691 | 10 | **GPDH** | 0.0150 | 9.5 | GAPDH | 10 |
| TUA | 1.344 | 11 | TUA | 1.535 | 11 | TUA | 0.0364 | 11 | TUA | 11 |
